# Transgenerational Defense Priming for Crop Protection against Plant Pathogens: A Hypothesis

**DOI:** 10.3389/fpls.2017.00696

**Published:** 2017-05-04

**Authors:** Gabriela Ramírez-Carrasco, Keren Martínez-Aguilar, Raúl Alvarez-Venegas

**Affiliations:** Centro de Investigación y de Estudios Avanzados del IPN, Unidad IrapuatoGuanajuato, Mexico

**Keywords:** priming, breeding programs, crops, epigenetics, transgenerational priming

## Abstract

Throughout evolution, plants have developed diverse mechanisms of defense that “prime” their innate immune system for more robust and active induction of defense responses against different types of stress. Nowadays there are numerous reports concerning the molecular bases of priming, as well as the generational priming mechanisms. Information concerning transgenerational priming, however, remains deficient. Some reports have indicated, nonetheless, that the priming status of a plant can be inherited to its offspring. Here, we show that the priming agent β-aminobutyric acid induced resistance to *Pseudomonas syringae* pv. phaseolicola infection in the common bean (*Phaseolus vulgaris* L.) We have analyzed the transgenerational patterns of gene expression of the *PvPR1* gene (*Phaseolus vulgaris PR1*), a highly responsive gene to priming, and show that a transgenerational priming response against pathogen attack can last for at least two generations. We hypothesize that a defense-resistant phenotype and easily identifiable, generational and transgenerational, “primed patterns” of gene expression are excellent indicators of the priming response in crop plants. Furthermore, we propose here that modern plant breeding methods and crop improvement efforts must include the use of elicitors to prime induced resistance in the field and, above all, to select for induced heritable states in progeny that is primed for defense.

## Introduction

Since the first Agricultural Revolution, humans have domesticated hundreds of plant species. Domestication of wild species of plants encompasses a variety of evolutionary changes that may decrease the fitness of a plant in the wild, but increase it under human exploitation. Ever since, and particularly after the Green Revolution, the selection of individuals with desirable alleles, the scrupulous breeding of high yielding genotypes, and a number of technological advances have allowed crop production to increase and to supply the nutritional requirements of the human population.

Many pathogens still affect crop production (including pathogenic bacteria, fungi, and viruses), however, and losses triggered by pests must be halted. Consequently, if farming is to support the human population for a long time into the future, additional sustainable strategies for crop production and improved integrated pest management systems (IPMS) must be developed.

Accordingly, crop protection plays an important role in maintaining crop productivity. Synthetic pesticides are a cost-effective way to control pests, but have many disadvantages (e.g., affect beneficial organisms, evolution of resistance to the pesticide, effects on human health and the environment, etc.) (Bruce et al., 2016). Alternative solutions to the use of pesticides include, for example, the development of new resistant crop cultivars, use of biological control agents, or the employment of novel plant activator agrochemicals that can be used to turn on natural plant defenses (Bruce, 2010).

Resistance elicitors, also known as plant activators or priming agents, are a class of agrochemicals that act by enhancing plant defenses against different types of stress (Conrath et al., 2002; Walters et al., 2005). Thus, priming of cells (Conrath et al., 2002, 2006; Conrath, 2009) can be induced by treatment with natural or synthetic compounds, including salicylic acid, 2,6-dichloroisonicotinic acid (Kauss et al., 1992), benzothiadiazole (Katz et al., 1998), or β-aminobutyric acid (BABA; Oostendorp et al., 2001). Priming results in a faster and stronger induction of plant defense responses and enhanced resistance to biotic or abiotic stresses in comparison to that found in unprimed plants exposed to the same stress (Conrath et al., 2002; Conrath, 2011).

Given that priming provides a long-lasting, broad-spectrum resistance to stress, it has been suggested that priming of plant defense is a promising alternative approach in modern disease management because “it could provide an effective mechanism for crop protection in the field” (Beckers and Conrath, 2007). Plant activators do not have direct toxic effects on the target organism, are compatible with IPMS, and can enhance biocontrol techniques (Bruce, 2010). Furthermore, the use of natural or synthetic resistance elicitors to induce plant immunity is now becoming commercially attractive, particularly because chemical control employing pesticides is turning out to be unsustainable and undesirable (Roberts and Taylor, 2016).

Recent progress has been made in comprehending the molecular basis of priming. For example, chemically induced priming in *Arabidopsis* is associated with the accumulation of inactive mitogen-activated protein kinases (Beckers et al., 2009). Priming has also been linked to di- or tri-methylation at lysine 4 of histone H3 (H3K4me2 and H3K4me3, respectively) and to lysine acetylation of histone H3 at lysine 9 (H3K9) or at lysine 5, 8, or 12 of histone H4 (H4K5, H4K8, and H4K12, respectively) in the promoter regions of defense-related genes (Jaskiewicz et al., 2011).

Regardless of ample reports concerning generational priming (reviewed in Cohen et al., 2016), information concerning transgenerational priming remains deficient. Some reports have indicated, however, that the priming status of a plant can be inherited to its offspring (transgenerational priming). For example, progeny of *Arabidopsis thaliana* plants that had been either primed with BABA or inoculated with *Pseudomonas syringae* pv *tomato* (avrRpt2) showed enhanced resistance to *P. syringae* pv *tomato* DC3000 (PstDC3000) and *Hyaloperonospora arabidopsidis*, as well as enhanced expression of defense-related genes (Slaughter et al., 2012). Additionally, systemic acquired resistance in *Arabidopsis* has been shown to be inherited epigenetically after inoculation with PstDC3000 (Luna et al., 2012). Furthermore, Rasmann et al. (2012) have shown that herbivory or mechanical damage produce progeny that is primed to express jasmonic acid-dependent defense responses in both *A. thaliana* and tomato (*Solanum lycopersicum*).

The examples listed above suggest that the transgenerational inheritance of defense priming mechanisms have an epigenetic component that could grant an adaptive benefit to the next generation. Indeed, small RNAs are needed for transgenerational resistance in *Arabidopsis* (Rasmann et al., 2012). In addition, transgenerational priming in *Arabidopsis* is transmitted by DNA hypomethylation in genes that lead priming of salicylic acid-dependent defense responses in the following generations (Luna et al., 2012). Furthermore, histone H3 lysine acetylation patterns (Luna et al., 2012) or histone H3 lysine methylation patterns (Martínez-Aguilar et al., 2016) at the promoter regions of defense genes generate primed states that could be transmitted to their descendants.

As in many other plants (Balmer et al., 2015; Cohen et al., 2016), resistance to pathogen infection (e.g., *P. syringae* pv. phaseolicola) in the common bean (*Phaseolus vulgaris* L.) can be induced with priming activators (Martínez-Aguilar et al., 2016). There are only a few studies of priming, however, in relation to the interaction of the common bean with its pathogens (Siegrist et al., 1997; Martínez-Aguilar et al., 2016) and nothing is known regarding the impact on plant defense responses in bean progeny from primed plants that have been exposed to pathogens. The common bean is an important crop worldwide, the most important grain legume for human consumption in the world, and an excellent model crop plant with which to study the transgenerational plant defense responses in progeny from primed plants that have been exposed to pathogens.

The aim of this work is to support the creation of a crop innovation pipeline to help plant researchers to assess and study, through easily-identifiable patterns of gene expression, key mechanisms of transgenerational defense priming. In turn, this will allow the generation of new technologies, methodologies, and crop improvement strategies with which to develop new crop varieties that are better suited to modern agriculture. We argue that crop improvement efforts must include the use of elicitors to prime or activate induced resistance in the field and, above all, to select for induced heritable epigenetics states in progeny that is primed for defense. This is particularly important considering that some quantitative trait loci used by breeders could occur due to epigenetic variation, instead of genetic variation (Springer, 2013). This study also opens the way to a new understanding of priming and epigenetics as a critical component in plant–pathogen interactions and in plant developmental processes.

## Experimentation

**FIGURE 1:**
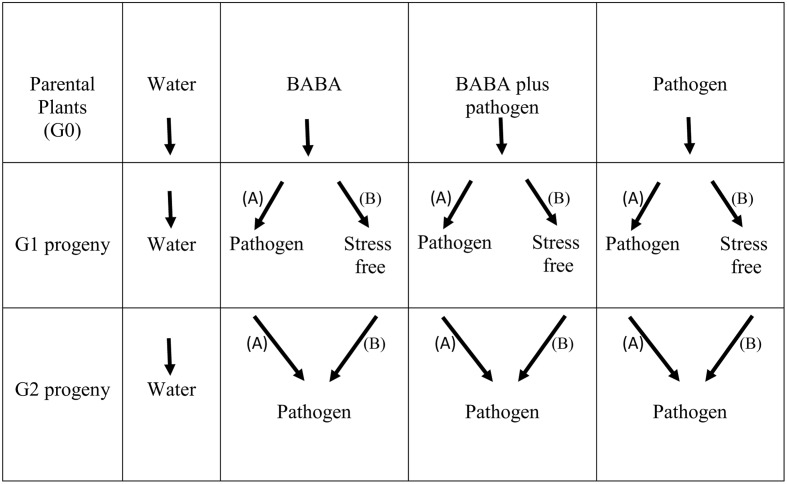
**Outline of the experimental design used in the present study**.

Previously, we explored the generational priming phenomenon and BABA-induced resistance in the common bean (cultivar BAT93) against *P. syringae* pv. phaseolicola NPS3121 (PspNPS3121) (Martínez-Aguilar et al., 2016). Accordingly, all the protocols have already been described (Martínez-Aguilar et al., 2016) and are therefore not presented here. For the transgenerational priming analysis shown here, however, all parental plants (G0 generation, cultivar BAT93) were self-pollinated (water only, BABA only, BABA plus pathogen, or pathogen only treated plants) and grown to set seed to generate G1 progeny lines (**Figure 1**). To determine the transgenerational priming, seeds from the G1 progenies (water only, BABA only, BABA plus pathogen, or pathogen only) were separated into two groups: (i) Group “A” were G1 progeny lines exposed only to the pathogen (PspNPS3121), without activator treatment (“continuous-stress generation”), giving G2 progeny seeds; and (ii) Group “B” were G1 progeny lines allowed to set seed under stress-free conditions (or, “stress-free generation”), giving G2 progeny seeds (**Figure 1**).

G2 progeny seeds (BABA only, BABA plus pathogen, and pathogen only) from both “A” and “B” G1 groups were grown and inoculated with the pathogen (PspNPS3121), as previously described (Martínez-Aguilar et al., 2016). Positive control parental lines and control progeny lines were not inoculated with the pathogen nor treated with BABA (“water only” progeny), but were allowed to set seed and used to normalize the data (**Figure 1**).

All G2 plants and G1 group “A” plants were inoculated with the pathogen at the same age (or developmental stage) as the parental lines were infected (17 days after germination, dag). Samples from challenged plants were taken from distal leaves that had not been exposed to the pathogen 24 h before infection, and 24 h and 120 h after infection (or 16, 18, and 22 dag, respectively).

Samples from the G1 group “B” plants, not treated with the pathogen, were taken at the same age as the infected plants (24 h before infection, and 24 and 120 h after infection; or 16, 18, and 22 dag, respectively).

RNA extraction, cDNA synthesis, and q-PCR conditions have been described previously (Martínez-Aguilar et al., 2016). The results presented here are from three independent biological replicates. Each biological replicate was tested in triplicate and q-PCR data were normalized to the Elongation Factor 1-α (*PvEF1α*) reference gene. The primers used were as follows: for the *P. vulgaris PvPR1* gene (*Phvul.006G196900*), Forward 5′-cacaaaactcaccccaagacttcctcaa-3′ and Reverse 5′-ttgcatcccatctcattggtcctacc-3′; and for the Elongation Factor 1-α (*PvEF1α*) reference gene, Forward 5′-ggtcattggtcatgtcgactctgg-3′ and Reverse 5′-gcacccaggcatacttgaatgacc-3′ (Barraza et al., 2015; Martínez-Aguilar et al., 2016).

### Effect of the Pre-challenge Priming Stage on Defense Priming against *P. syringae* pv. Phaseolicola

**FIGURE 2:**
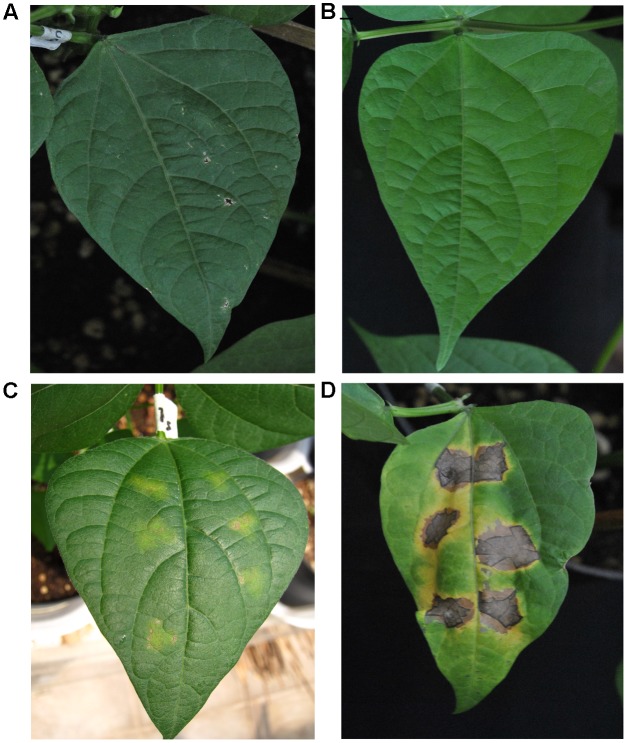
**Lesion development in leaves from *Phaseolus vulgaris* parental plants (G0) inoculated with *Pseudomonas syringae* pv. phaseolicola NPS 3121 (PspNPS3121) after treatment with: (A)** water, non-inoculated; **(B)** only BABA, non-inoculated; **(C)** BABA plus PspNPS3121; **(D)** only PspNPS3121. Photos were taken 10 days after pathogen inoculation.

Our first goal was to establish, once again, that BABA primes common bean plants for resistance to the PspNPS3121 pathogen. The BABA priming stimulus before pathogen infection resulted in a significant resistance, after pathogen infection, against PspNPS3121 (**Figure 2**). This type of phenotype is an excellent indicator of the protection provided by the priming activators against spreading of the pathogen, and should be the first step to consider in the pipeline for crop innovation by generational and transgenerational defense priming. Even if there is not a clear or strong visible phenotype, however, there is still an induction of plant defenses in the form of, for example, transcript accumulation of defense genes (as we have assessed with other tolerant and resistant cultivars to the pathogen; data not shown), or even metabolites and chromatin changes.

**FIGURE 3:**
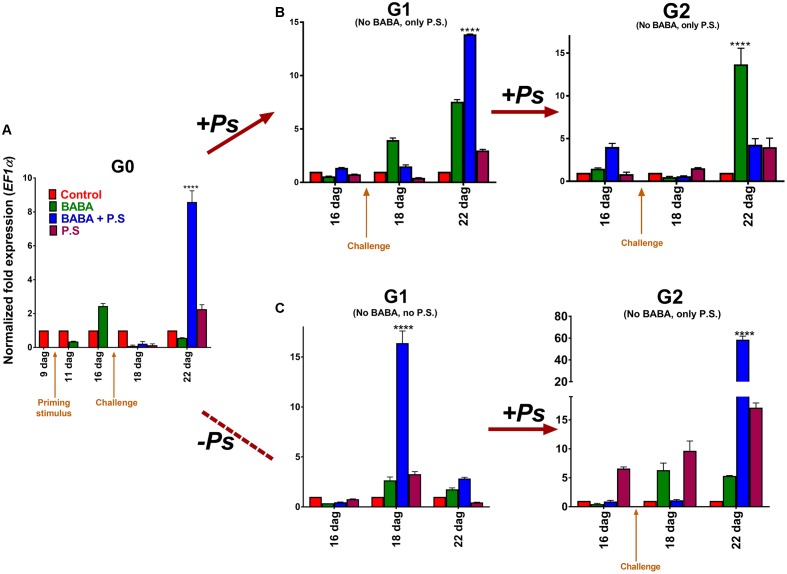
**Transcript levels of *PvPR1* from *Phaseolus vulgaris* as determined by qRT-PCR at various days after germination (dag)**. **(A)** Foliage leaves, or first true leaves, from 10-day-old plants, were BABA-treated and samples, from distal leaves that had not been directly exposed to the activator, were taken 24 h before (9 dag), and 24 h after (11 dag) treatment. G0 plants were given one of three treatments: primed with the activator (BABA), followed by inoculation with *Pseudomonas syringae* pv. phaseolicola (BABA + P.s.; infected at 17 dag); inoculated only (no activator + P.s.; infected at 17 dag); or primed only (BABA + –). Samples were taken from distal leaves that had not been exposed to the pathogen 24 h before infection, and 24 and 120 h after infection (or 16, 18, and 22 dag, respectively). **(B)** G1 plants (from G0 BABA only, BABA plus pathogen, and pathogen only) were exposed only to the pathogen (PspNPS3121), without activator treatment (“continuous-stress generation”), giving G2 progeny seeds. G2 progeny seeds (BABA only, BABA plus pathogen, and pathogen only) were grown and were inoculated with the pathogen (PspNPS3121). **(C)** G1 plants (from G0 BABA only, BABA plus pathogen, and pathogen only) were not exposed to the pathogen (“stress-free generation”) and did not undergo activator treatment, giving G2 progeny seeds. G2 progeny seeds (BABA only, BABA plus pathogen, and pathogen only) were grown and inoculated with the pathogen (PspNPS3121). Data were normalized to the elongation factor 1-α (*PvEF1α*) reference gene. Data represent mean ± SD, *n* = 3 independent experiments. Statistical significance was determined with multiple Student’s *t*-tests, followed by the Holm–Šídák multiple comparison test at a significance value of 0.05, by using the GraphPad Prism (v 6.0, GraphPad Software, San Diego California USA, www.graphpad.com). Abbreviations: dag = days after germination; BABA = β-aminobutyric acid; P.S. = *Pseudomonas syringae* pv. phaseolicola NPS 3121.

Next, to investigate the effect of priming on transcript levels, we used real time PCR (q-PCR) to monitor transcript accumulation of the *PvPR1* gene (*Phaseolus vulgaris PR1*), the gene ortholog to the *Arabidopsis PATHOGENESIS RELATED GENE-1* (*PR-1*), and a very responsive gene to priming (Slaughter et al., 2012). Transcript accumulation of *PvPR1*, in the G0 generation, showed the characteristic biphasic curve distinctive of the priming response (**Figure 3A**; Balmer et al., 2015). That is, the activator did not trigger major changes in gene expression during the priming phase, but left the plants in a standby state. After inoculation of the BABA-primed plants with PspNPS3121, however, transcripts were induced rapidly and accumulated at significantly higher levels (∼ 9-fold) than in the unprimed, inoculated controls. In non-infected BABA-treated plants, conversely, transcript levels of *PvPR1* tend to decrease with time in the absence of challenge (**Figure 3A**). These results corroborate that chemical inducers, such as BABA, promote defense priming (Cohen et al., 2016). Thus, a defense-resistant phenotype and “primed patterns” of gene expression are excellent indicators of the generational priming response in crop plants.

Accordingly, the inheritance of the induced defense resistance will depend on the kind of stress, the age of the plant at the activation of priming and exposure to the pathogen, the concentration and type of priming agent to which the parental lines were exposed, and the specific crop species and cultivars under consideration.

### Priming Under Continuous Stress

To analyze the transgenerational priming response, we challenged two-week-old plants from the G1 progeny (from G0 primed, inoculated plants; primed, non-inoculated plants; and unprimed, inoculated plants) with PspNPS3121 (without application of the priming activator), and compared transcript accumulation of *PvPR1* to control unprimed, non-inoculated plants, 5 days later (**Figure 3B**). Pathogen inoculation induced elevated transcript levels in G1 plants from parents that had been primed and inoculated, revealing a memory of the treatment to which they were subjected. This result illustrates a transgenerational priming effect, where the plants were able to react more rapidly and more efficiently when challenged with the pathogen. Additionally, enhanced transcription accumulation for *PvPR1* also took place five days after inoculation in G1 plants from parents that had been only primed with BABA (although to a lesser extent than primed and inoculated plants). This suggests that BABA-primed plants can have a memory of the priming treatment and a transgenerational response to pathogen attack.

Next, to determine if the transgenerational priming response can persist for more than one generation under continuous stress, we challenged 2-week-old plants from the G2 progeny (from G1 challenged plants coming from G0 primed, inoculated plants; primed, non-inoculated plants; and unprimed, inoculated plants) with PspNPS3121 (without application of the priming activator), and compared transcript accumulation of *PvPR1* to control unprimed, non-inoculated plants, 5 days later (**Figure 3B**). The transgenerational priming response was lost in the G2 generation, for descendants of common bean plants that were primed and inoculated in the G0, and that were challenged in the G1 generation. Clearly, this effect will be influenced by the crop species and cultivars under consideration; however, this result gives an indication of the approaches to follow during crop improvement, of the crop species and cultivars to assess, and of the possible outcomes.

Plants that had been BABA-primed (G0) and continuously challenged (G1, G2), however, showed increased transcript accumulation of the *PvPR1* defense-related gene. In other words, they exhibited a defense memory, since the information of the priming stimulus was stored until a triggering stimulus activated gene expression (Martinez-Medina et al., 2016). Thus, BABA-treated plants were sensitized to additional treatments. Basically, the continuous challenge kept the priming memory, which suggests that progeny from non-challenged but primed plants can possess transgenerational priming responses against pathogen attack for at least two generations.

### Priming Under Non-continuous Stress

To analyze the transgenerational priming effect under non-continuous stress conditions and to establish that transgenerational priming is not exclusively perceived following immediate BABA-activation, we analyzed *PvPR1* gene expression in 2-week-old stress-free plants from the G1 progeny (from G0 primed, inoculated plants; primed, non-inoculated plants; unprimed, inoculated plants; and unprimed, non-inoculated plants). Stress-free G1 plants that were primed and inoculated in the G0 showed elevated expression levels of the *PvPR1* “marker” gene (**Figure 3C**). These expression levels, interestingly, peaked in the G1 progeny at the same developmental stage, or at the same time, as when the parental (G0) plants were inoculated with the pathogen. In other words, primed and challenged plants displayed a “transgenerational memory response” to pathogen attack, even in the absence of actual pathogen challenge. Thus, primed plants exposed to the pathogen can transfer their competence against the encountered stress to their progeny. Since the G1 plants were not challenged, however, *PvPR1* expression levels quickly tended to decrease, as in the pre-challenge priming phase of the different priming states (as described by Balmer et al., 2015).

Next, we collected and germinated seeds from the G1 stress-free progeny, and then challenged 2-week-old plants from the G2 progeny (from non-challenged G1 plants coming from G0 primed, inoculated plants; primed, non-inoculated plants; and unprimed, inoculated plants), with PspNPS3121 (without application of the priming activator). The transgenerational priming response was re-established in the G2 generation, as determined by enhanced transcription accumulation of *PvPR1*, 5 days after pathogen inoculation of G2 plants (**Figure 3C**). Specifically, transcript accumulation was higher in plants that had been primed and challenged (from G0 primed, inoculated plants), came from a stress-free generation (non-challenged G1 plants coming from G0 primed, inoculated plants), and had been challenged again in the G2 generation. This result indicates a transgenerational priming response against pathogen attack that can last for at least two generations.

In addition, G2 non-primed but inoculated plants that were challenged in the F0 and non-challenged in the G1, showed increased transcript accumulation of the *PvPR1* gene (24 and 120 h after pathogen inoculation) when compared to the progeny of non-primed plants. This suggests that G2 progeny from G0 challenged but non-primed plants can possess transgenerational responses against pathogen attack even if the plants experience a stress-free generation (although to a lesser extent than BABA-primed plants). In other words, they exhibited a type of “defense memory”, given that the information of the stimulus was stored until a new triggering stimulus activated gene expression (Martinez-Medina et al., 2016). In contrast, G2 non-primed but inoculated plants that were primed but non-challenged in the G0 and non-challenged in the G1, showed a decrease in *PvPR1* transcript accumulation, suggesting that after a stress-free generation their transgenerational defense memory is lost. That is, BABA-primed (G0) plants require to be continuously challenged (G1, G2), in order to display a transgenerational defense memory and enhanced transcript accumulation of the *PvPR1* defense-related gene.

It will be essential, however, to determine if the transgenerational memory will persist in non-challenged G3 progeny and subsequent generations as in the G1 generation, or if by challenging the G3 progeny the transgenerational priming response will be lost, as seen in plants under continuous stress. Such a patterns of gene expression must be considered in breeding programs when choosing for cultivars with desirable phenotypic traits to develop novel resistant cultivars.

### Breeding New Resistant Crop Cultivars

During the last decades, modern breeding methods, novel research, and agricultural intensification have produced many beneficial traits in crop species. Because of population growth, however, global food production must increase to support the human population for years to come. Future reliance on an increased use of fertilizers, plant growth regulators, and pesticides would be dangerous. Thus, it is imperative to reduce the impacts of pests and diseases on crop yields, to develop further improvements to crop species, and to translate fundamental plant science research into new crop varieties. Furthermore, we must identify the mechanisms by which epigenetic variation may modify plant gene regulation and phenotype, and we should focus on how the epigenome acts as a strong new source of diversity for agronomically important traits and its potential for exploitation in crop improvement programs.

As suggested by Rhee et al. (2016), crop genome complexity is partly due to the expansion and diversification of disease *Resistance* (*R*) genes. Furthermore, improved disease resistance has mainly depended on the introduction of *R* genes into high-yielding crop varieties. Considering that there is a limited scope for “breeding for higher yield due to the restricted genetic potential of crops to increase overall production” (Bruce, 2010), an augmented and heritable induction of plant defenses, as well as selecting for inducible epialleles that contribute to desired traits, is highly desirable for crop production and can be extremely effective against pathogen attack, particularly when the activation of the defense mechanisms (e.g., gene expression) precedes such stress. Accordingly, priming activators could complement this, as they are fully compatible and offer an alternative to synthetic pesticides, biological control, and other forms of control within integrated disease management systems.

As presented here, treatment of bean plants with BABA induces a primed state, characterized as a faster and stronger transcript accumulation of the *PvPR1* plant defense gene, which is transmitted to the progeny. However, it remains to be explored whether all crop species behave in a similar way (or under what conditions) or whether this response is exclusive to some distinct cultivars (e.g., there are about 36,000 accessions of *Phaseolus* spp., corresponding to 44 taxa from 112 countries)^1^.

Inheritance of the induced state, or priming status, clearly has an epigenetic component (transmission of small RNAs, Rasmann et al., 2012; DNA hypomethylation, Luna et al., 2012; chromatin remodeling, Jaskiewicz et al., 2011; histone modifications, Martínez-Aguilar et al., 2016); however, each of the epigenetic states must be determined experimentally (e.g., DNA methylation, histone methylation/acetylation, chromatin remodeling, etc.) for every crop and many specific conditions (activator type; time and mode of application; laboratory, greenhouse, or field conditions; etc.), to assess the long-term impact on gene expression and plant immunity.

In reality, epigenetics must have contributed to the heritable natural variation that has been selected during plant breeding and crop improvement, and important quantitative trait loci (QTL) exploited by plant breeders correspond to epigenetic variation, rather than to genetic variation (Springer, 2013). Furthermore, as suggested by Richards (2011), epigenetic variants can be used as parents in methodical crosses and pedigree analysis, to identify and separate the genetic and epigenetic components of such QTL (Richards, 2011).

As revealed by Slaughter et al. (2012), the capacity for transgenerational priming is not accession specific, in the case of *A. thaliana*. Additionally, the observation of inherited resistance in different species (Luna et al., 2012; Slaughter et al., 2012; Lankinen et al., 2016; Martínez-Aguilar et al., 2016) suggests that this trait may be more widely distributed in plants. Consequently, one way to accelerate the development of long-lasting pathogen resistance is to activate and select for primed resistance genes. The priming state of the plants at the molecular level, displayed by the levels of transcript accumulation of defense genes, is an excellent indication of the potential that a cultivar possesses, as well as an opportunity to select for new cultivars in breeding programs. Specifically, it will be important to select for cultivars that display patterns of expression of “marker” genes similar to those presented here; that is, primed progeny under continuous stress with enhanced levels of expression as a transgenerational response to pathogen attack (**Figure 3B**), or primed progeny under stress-free conditions with enhanced gene expression as a result of a transgenerational defense “memory” mechanism (**Figure 3C**).

The level of primed resistance of the descendants, accumulated during subsequent generation(s), must therefore be continuously tested to identify greater plant resistance and enhanced gene expression of defense-related genes according to the experimental conditions. In field experiments, a combination of two or more elicitors can be used to develop synergistic activity against pathogens. Additionally, plant activators can be combined with pesticides to provide enhanced disease control and increased yields.

Once the primed varieties resistant to the disease are selected, it will be promising to perform prime-omics analysis to elucidate the overall priming process (Balmer et al., 2015); that is, analysis of transcriptional, proteomic, and/or metabolic data to display the priming of crop plants.

It is important to emphasize that the molecular and epigenetic mechanisms that underlie induced transgenerational priming may depend on a number of factors, including plant species, type of stress, severity of the disease, environmental conditions, form of application, and type of priming agent to which parental lines were exposed. Accordingly, plant breeding programs must be oriented to select, under an optimal combination of parameters, for novel/significant traits that may improve the level of disease resistance response concomitant with a reduction of costs (e.g., negative impacts on plant growth, crop productivity, etc.). Furthermore, the transgenerational priming response should be investigated not only against other biotrophic, hemi-biotrophic, and/or necrotrophic pathogens, but also against viruses and abiotic stress. A fine-tuned comprehension of how plants can use this memory, and in what situations, is of great interest and must be employed to increase crop productivity.

Thus, modern plant breeding methods and crop improvement efforts must include the use of elicitors to prime or activate induced resistance in the field and, above all, to select for induced heritable epigenetics states in progeny that is primed for defense, in combination with single nucleotide polymorphisms or other genetic markers, particularly when considering that quantitative traits are controlled by multiple loci. Moreover, the use of priming activators must be a key component in integrated pest management programs to help reduce, for example, the use of pesticides (a threat to natural ecosystems and biodiversity).

## Concluding Remarks

In this paper, we addressed the importance of priming as a promising approach for crop protection. This is based on the assumptions that generational and transgenerational priming can be easily recognized by analyzing the expression patterns of responsive genes and examining the resistance phenotype of primed plants after being challenged. By examining the patterns of gene expression in the progeny of primed plants, under different stress conditions, it will then be possible to select and develop novel resistant cultivars with desirable phenotypic traits or improved defense responses. Even though we have chosen to analyze the *PvPR1* “marker” gene, there remain an unidentified number of responsive defense genes, metabolites, and epigenetic modifications that must be analyzed for their connection to the priming phenomenon during the priming events.

A potential disadvantage of priming for crop improvement is that, under specific circumstances, the epigenetic states may not be transmitted to the progeny, or they could display partial heritability within a population. This, however, creates the opportunity to, for example, explore novel natural priming activators, analyze new epialleles, or explore new combinations of treatments to prime and turn on natural plant defenses. In addition, induced resistance may not provide the “normal” level of protection that we usually observe after the application of pesticides; however, priming can be used in combination with pesticides, biological control, resistance breeding, or any other integrated pest management strategy. Furthermore, since induced resistance by means of priming involves multiple defenses (“multigenetic trait”, Roberts and Taylor) and is regulated by numerous plant defense genes (“quantitative resistance”, Bruce et al., 2016), it is a lasting form of pathogen protection with a negligible probability of pathogens surmounting resistance. In addition to this, the activation of priming and the selection of cultivars with a transgenerational defense priming holds many benefits to breeding programs for the development of beneficial new traits in crops.

The information presented here has the potential to help plant researchers to assess and study easily identifiable key mechanisms of transgenerational defense priming and select for new straightforwardly primed cultivars to be used in breeding programs.

## Author Contributions

RA-V provided the idea of the work. RA-V, GR-C, and KM-A designed the experiments. KM-A and GR-C conducted the phenotypic analysis and qPCR. RA-V, GR-C, and KM-A participated in the interpretation of results and critically reviewed the manuscript. RA-V wrote the paper. All authors read and approved the final manuscript.

## Conflict of Interest Statement

The authors declare that the research was conducted in the absence of any commercial or financial relationships that could be construed as a potential conflict of interest.

## Footnotes

1 http://ciat.cgiar.org/what-we-do/crop-conservation-and-use/bean-diversity/
